# Do economic evaluation studies inform effective healthcare resource allocation in Iran? A critical review of the literature

**DOI:** 10.1186/1478-7547-12-15

**Published:** 2014-07-11

**Authors:** Hassan Haghparast-Bidgoli, Aliasghar Ahmad Kiadaliri, Jolene Skordis-Worrall

**Affiliations:** 1 Institute for Global Health, University College London, London, UK; 2 Health Economics Unit, Department of Clinical Sciences-Malmö, Lund University, Lund, Sweden; 3 Department of Health Management and Economics, School of Public Health, Tehran University of Medical Sciences, Tehran, Iran; 4 Department for Global Health and Development, London School of Hygiene and Tropical Medicine, London, UK

**Keywords:** Economic evaluation, Pharmacoeconomics, Review, Iran

## Abstract

To aid informed health sector decision-making, data from sufficient high quality economic evaluations must be available to policy makers. To date, no known study has analysed the quantity and quality of available Iranian economic evaluation studies. This study aimed to assess the quantity, quality and targeting of economic evaluation studies conducted in the Iranian context.

The study systematically reviewed full economic evaluation studies (n = 30) published between 1999 and 2012 in international and local journals. The findings of the review indicate that although the literature on economic evaluation in Iran is growing, these evaluations were of poor quality and suffer from several major methodological flaws. Furthermore, the review reveals that economic evaluation studies have not addressed the major health problems in Iran.

While the availability of evidence is no guarantee that it will be used to aid decision-making, the absence of evidence will certainly preclude its use. Considering the deficiencies in the data identified by this review, current economic evaluations cannot be a useful source of information for decision makers in Iran. To improve the quality and overall usefulness of economic evaluations we would recommend; 1) developing clear national guidelines for the conduct of economic evaluations, 2) highlighting priority areas where information from such studies would be most useful and 3) training researchers and policy makers in the calculation and use of economic evaluation data.

## Review

### Introduction

Economic evaluations identify, measure, value and compare the cost and consequences of two or more alternative programs or interventions [1]. Economic evaluation is commonly used as a decision tool in health care systems where, due to resource constrains, policy-makers have to choose between alternative activities with different implications for resources allocation [2].

Iran is a middle-income country with a population of 76 million. In 2009, Iran had a Gross National Income (GNI) per capita of US$ 10,250 and approximately 6% of its gross domestic production (GDP) per capita is spent on healthcare [3]. The constitution emphasizes the right of access to the highest level of health for all citizens and the Ministry of Health and Medical Education (MOHME) is responsible for fulfilling this goal through designing and implementing national level policies. There is at least one medical university in every province and at the provincial level, these universities play an important role in the provision of health services and medical education. The Dean of a medical university is the highest health authority at the province, reporting to the MOHME [4]. Health services are provided by public, quasi-public and philanthropic organizations, and a large network of private providers. The public sector (lead by MOHME) is the main provider of Primary Health Care across the country and provides a large part of secondary and tertiary health services [4]. The private sector mainly provides secondary and tertiary services in urban areas. Nearly 90% of the country’s population is covered by health insurance, mainly public health insurance organizations [4,5]. In spite of the high insurance coverage rate, health services (secondary and tertiary) are not affordable for many due to high out-of-pocket expenditures (mainly form of co-payments) [5]. In 2011, out-of-pocket expenditure was 60% of total health expenditures [3].

Iran’s health expenditure has been increasing rapidly over the last decade [5]. This trend is being driven by an ageing population, a rising prevalence of non-communicable and chronic diseases, an increase in the use of health technologies, a rise in domestic drug manufacturing and increasing prescription and consumption of medicines [6-8]. At the same time, the Iranian health system is being criticized for inefficient resource allocation, providing services without cost-effectiveness considerations, failing to regulate the private sector and over-utilization of new technologies [9-12]. Resource allocation decisions in Iran’s healthcare system, have historically been based on implicit criteria such as pre-existing service availability, affordability of the insurance organisations and providers and political pressure [5,12]. Recently, interest in evaluating efficiency including estimating efficiency of health care providers [6-8] and economic evaluation of interventions and new technologies has been growing in Iran, like many other Asian countries [13]. The government and MOHME have developed various strategies to improve both the efficiency and equity of resource allocation, including; establishing the Ministry of Welfare and Social Security (MWSS) in 2005, incorporating all health insurance organisations under MWSS with the aim of separating the health care providers/MOHME from the financiers [5,11], and establishing the Technology Assessment Unit within MOHME in 2007 [14]. Despite these efforts, the extent to which evidence from economic analyses is used to inform resource allocation in national strategic planning and decision-making remains unclear [5,12,15].

Despite an increase in the number of economic evaluation studies worldwide, concern about the quality of these studies, among other factors, has been one of the major barriers which limit their application by policy-makers [16-18]. In response to this policy concern, several guidelines have been developed to assess the quality of economic evaluation studies [1,19,20] and many systematic review studies has been conducted in different countries [21-25]. Arguably, these reviews focus disproportionately on high-income countries.

While a number of economic evaluations have been conducted in Iran, no known study has systematically reviewed the quality of Iranian economic evaluations. The purpose of this study was to provide a review of the state of economic evaluation within the context of Iran. Specifically, this review assessed whether Iranian economic evaluation studies had been performed according to current international standards and therefore whether their results are likely to prove useful to policy makers. Moreover, this study examined whether these studies were aimed at those conditions generating a higher burden of disease in Iran.

## Methods

### Literature search and study selection

A literature search was conducted independently by the first author in December 2011 and then verified by the second author and updated in May 2012. The search was performed using the following international databases: Medline/Pubmed, Embase, Web of Science, NHS Economic Evaluation Database (EED), Econlit and Google scholar. To identify articles published in national journals (both in Persian and English), the Scientific Information Database (SID) website was searched. In addition, the references of retrieved articles were manually searched for further papers. The search was continued until no new articles were found. The keywords used for the literature search were: “cost analysis”, “cost*” “economic evaluation”, “cost-effective”, “cost-saving”, “cost-effectiveness”, “cost-utility”, “cost-benefit”, “cost-minimisation” and “Iran*” in the title or abstract of the articles. The full search strategy is available in Additional file 1. Studies were included in the review on the basis of the following inclusion criteria:

• Full economic evaluation, i.e., comparative analysis of costs and outcomes of at least two interventions [1] (e.g., cost minimization analysis [CMA], cost-effectiveness analysis [CEA], cost-utility analysis [CUA] or cost-benefit analysis [CBA]);

• Studies used primary or secondary data;

• Original articles published in international and Iranian journals;

• Published in English and Persian (Farsi) languages;

• Applied to the Iranian context;

• Published prior to (June) 2012.

### Data extraction and critical appraisal

Relevant papers were selected by screening the titles and abstracts (first step) and entire articles (second step) according to the inclusion criteria listed above. Screening of articles was conducted independently by HHB and AAK. Any disagreements about eligibility between the authors were solved through subsequent discussion with JSW.

Assessment of the quality of included studies was done using a questionnaire adapted from existing guidelines, checklists and other review articles of economic evaluations [1,19,20,24,26]. The questionnaire included both general information and economic evaluation features of the selected articles. General information included: year of publication, journal in which the study was published (national or international), affiliation of the first author (medical or non-medical), type of journal (medical, non-medical), language (English or Persian) and geographical location covered by the study. The main economic evaluation features of the questionnaire included method of economic evaluation (CMA, CEA, CUA, CBA), study design, perspective (both stated and evaluated), type of sensitivity analysis performed, time horizon, type of outcome and its description, disease investigated, intervention type, description of intervention (competitors), type of data used (primary or secondary), types of costs included and if they were measured and valued properly, sample size, funding source, whether economic evaluation was the primary study goal, whether discounting were performed (if the costs and/or outcomes were from a study period of >1 year) and whether generalisability of results was discussed. Both first and second authors reviewed the selected articles independently and extracted the information into predesigned forms in Excel. Any disagreements were solved through subsequent discussion.

To investigate whether the published studies targeted high priority interventions, a study of the burden of disease and injuries in Iran was used to identify high burden health problems in the country [6]. The study, which used methods developed by the World Health Organization (WHO) [27] for national burden of disease (NBD) studies, measured disease burden in terms of disability-adjusted life years (DALYs) in the Iranian population in 2003. In any resource constrained environment, economic evaluation studies should ideally provide evidence on cost-effectiveness of interventions to address diseases with a high burden [2]. Therefore, analysing the disease burden and available evidence from economic evaluation studies would provide useful information for researchers and policy makers to establish gaps in knowledge and priorities for future study. This same approach has been previously used by a number of other researchers [2,28,29].

## Results

**Figure 1:**
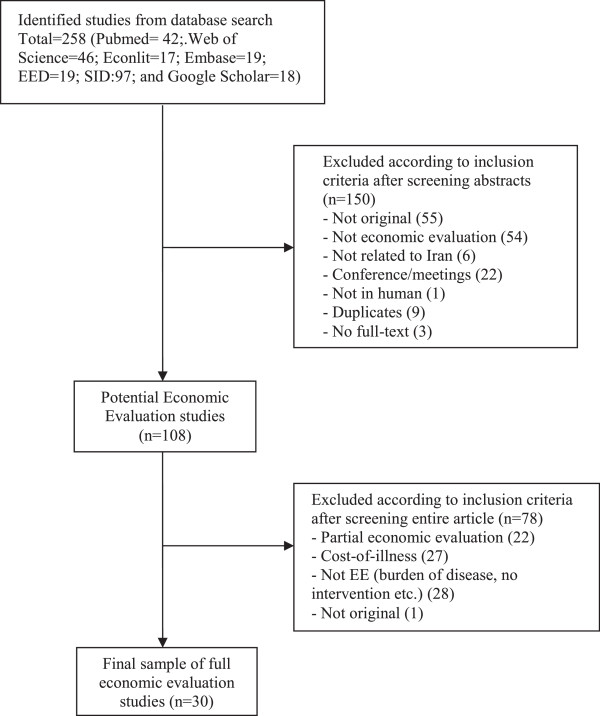
Flowchart of article selection.

A total of 258 articles were identified by the literature search, of which 150 articles were excluded at the initial stage because they violated basic inclusion criteria (Figure 1). The full-texts of 108 articles were then reviewed and a further 78 articles were dropped because they failed one or more inclusion criteria. The remaining 30 articles [30-59] met the inclusion criteria of the study. Details of the reviewed studies are presented in Additional file 2: Table S1 and Additional file 3: Table S2.

### General characteristics of included studies

**Figure 2:**
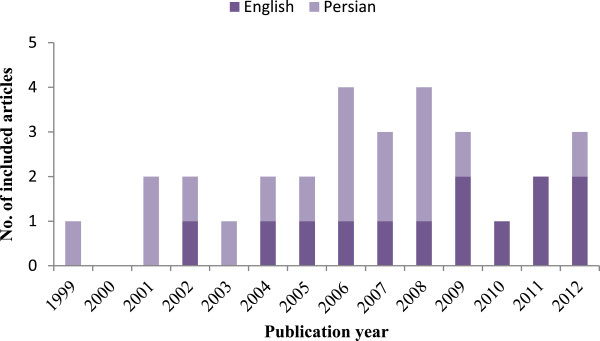
Included studies by language and publication year.

As can be seen from the Figure 2, the numbers of full economic evaluation papers are scarce. Of the included articles, 17 (57%) were published in Persian. General characteristics of the reviewed studies are presented in Table 1. The majority of the papers (77%) were published in medical journals rather than specialized health economics or health care sciences journals, and in twenty-two papers (73%) the first authors had medical/clinical affiliations. Most studies (85%) covered sub-national geographical locations (province level), and only five studies had national coverage (Table 1).

**Table 1:** General characteristics of the included studies

**Variable**	**Categories**	**N (%)**
Affiliation of the first author	Medical/Clinical	22 (73)
	Non-medical*	8 (27)
Where journal published	International	11 (37)
	Regional	2 (7)
	National	17 (57)
Type of journal	Medical	23 (77)
	Non-medical**	7 (23)
Language published	English	13 (43)
	Persian	17 (57)
Geographical location	National	5 (17)
	Sub-national	25 (83)

*Including epidemiology, health economics, health management etc.

**Including public health, health economics, health policy and management, economics etc.

### Economic features and quality of included studies

Table 2 reports economic and methodological features of the selected studies and Table 3 shows the extent to which these studies meet the recommendations for good reporting of economic evaluations. Of the 30 reviewed studies, 21 studies were CEA (70%). In terms of study design, most studies were observational (43%) following by randomized controlled trials (RCT) (27%). Only 6 studies (20%) employed a modelling approach, which among them, type of the model used was not specified in two studies [34,38]. While most studies did not specify their perspective (77%), we assessed the perspective of the studies, as shown in Table 2. The health care system/provider (77%) was the most frequent perspective. Two studies [30,58] stated their perspective as societal. The majority of the studies did not perform any sensitivity analysis (73%) and those that did so [30,32,36-39,47,58] mainly conducted one-way sensitivity analysis (17%).

**Table 2:** Economic features of the included studies

**Feature**	**N**	**%**
**Type of economic evaluation**		
CEA	21	70
CUA	5	17
CBA	4	13
**Study design**		
RCT	8	27
Quasi-experimental	3	10
Modelling	6	20
Observational (prospective, retrospective etc.)	13	43
**Perspective evaluated**		
Societal	0	0
Government	3	10
Healthcare system/Healthcare provider	23	77
Third party	2	7
Patients	1	3
Mixed	1	3
**Type of sensitivity analysis**		
One-way analysis	5	17
Multi-way analysis	1	3
Univariate/multivariate regression	1	3
Probabilistic analysis	1	3
Not performed	22	73
**Time horizon**		
<= 1 year	8	27
1-10 years	5	17
Over 10 years	5	17
Not specified	12	40
**Type of outcome**		
QALY/DALY	5	17
Intermediate (physiological, functional, etc.)	21	70
Monetary	4	13
**Level of care and intervention type**		
Primary prevention	2	7
Curative (Surgical/Medical procedure)	10	33
Curative (Pharmaceuticals)	7	23
Diagnostic/screening (secondary prevention)	10	33
Mode of delivery of care	1	3
**Type of data used**		
Primary data	22	73
Secondary data	4	13
Mixed	4	13
**Type of costs included**		
Direct medical costs	30	100
Direct non-medical costs	4	13
Indirect costs (Productivity loss)	0	0

**Table 3:** **Extent to which the published evaluations met recommendations for good reporting of economic evaluation studies **[1,20]

**Criteria**	**Number of studies fulfilling recommendation (n/N)**	**%**
Competing alternatives clearly described	18/30	60
Economic evaluation as primary objective	22/30	73
Time horizon stated	18/30	60
Perspective specified	7/30	23
All important and relevant costs for each alternative identified	9/30	30
All included cost measured appropriately	14/30	47
All included costs valued appropriately	12/30	40
Sources of cost data included	24/30	80
Sources of outcome data included	29/30	97
ICER/ BCR/NPV calculated and reported	8/30	27
Cost discounted	3/12	25
Outcome discounted	3/12	25
Sensitivity analysis performed	8/30	27
Generalisability of findings discussed	4/30	13
Funding sources disclosed	10/30	33

ICER: Incremental Cost-Effectiveness Ratio, BCR: Benefit-Cost Ratio, NPV: Net Present Value.

The analysis showed that 40% of the studies failed to report their time horizon clearly. Among those that did report the timeline, eight (27%) had time horizons of less than one year, five (17%) between 1 to 10 years and five over 10 years.

Among CUA studies (i.e. studies using comprehensive/composite outcome measures such as quality-adjusted life years [QALYs] or DALYs as their primary outcome measure) three studies used QALYs [35,37,38] and two DALYs [39,52] as the primary effectiveness measure. Among the three studies using QALYs, one used utility scores obtained from other countries [38].

Among CEA studies (i.e. studies using intermediate measures or natural units such as deaths prevented or cases detected as the primary outcome measure), the majority of studies used disease specific outcome measures such as mortality rates, number of patients detected or number of complications, and none generic measures such as “life years gained/saved”.

Only four CBA studies were performed in the context of Iran [41,42,48,56]. However, after evaluating these studies closely using the criteria recommended by Drummond, et al. [1] and Zarnke, et al. [18], these studies were not fully measured the benefits of the interventions under investigation. The benefits in these studies have been defined as cost savings (mainly savings in medical costs) without measuring and valuing the monetary values of health gained by the intervention.

More than half (56%) of the reviewed studies focused on curative services including surgical/medical procedures (10 studies, 33%) and pharmaceutical interventions (7 studies, 23%). Other studies evaluated screening/diagnostic procedures (10 studies, 33%) and primary preventive care (2 studies, 7%). Only one study (3%) evaluated a mode of delivery of care.

The source of cost and outcome data was clearly specified in the majority of studies (80% for costs and 97% for outcomes). In 73% of cases, cost and outcomes data were gathered through primary data collection. In those studies collecting primary data, the median sample size was 120, ranging from 30 to 1,165,169. Three studies used a hypothetical cohort [34,39,58] and three studies did not clearly specify their sample size [30,38,52]. Among those studies using secondary data (8 studies), cost data were usually collected from national surveys/reports or previous studies conducted in Iran. In four studies, outcomes data were obtained from studies conducted in settings other than Iran [30,32,34,38].

All studies included direct medical costs, however only four studies (13%) included direct non-medical costs [32,39,56,58]. Among the two studies that adopted a societal perspective [30,58], none estimated productivity loss and intangible costs. It is important to identify the relevant cost items for each intervention, to measure the resources used (in their physical units), and to value these resources (by their prices) properly. Only 9 studies (30%) identified all relevant costs related to each alternative intervention, considering their perspective. In most cases, only the costs of medicine and hospitalization were included and capital costs (such as building and equipment) and the cost of medical supplies were not often calculated. The data about hospitalisation costs were mainly extracted from patients’ hospital records or obtained from the patients’ hospital bills. Six studies [30,34,36,37,48,58] described clearly how they measured and valued their cost components. Out of the 12 studies in which the reported time horizon was more than one year, only two studies discounted both costs and outcomes at the same time [39,42]. A further two studies discounted either costs [30] or outcomes [48] only. The common discount rate utilised in the studies was 3%. Only 5 studies [32,35,36,38,39] calculated and reported incremental cost-effectiveness ratios (ICER). Three studies [41,42,48] reported benefit-cost ratios (BCR). Although discussing the generalizability of results to the national level or to other settings can be an important element of an economic evaluation, the majority of reviewed studies (87%) failed to do so. Only four studies discussed the issue of generalizability to other settings to some degree.

**Figure 3:**
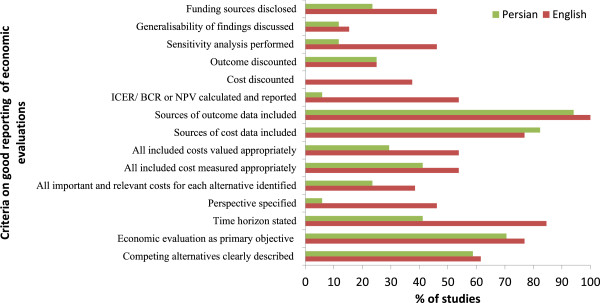
Comparing the criteria for good reporting of economic evaluations between English and Persian language studies.

Figure 3 compares the criteria for good reporting of economic evaluations. Based on the figure the English language studies, which were published in international journals, more often adhered to the recommendations for good reporting of economic evaluations compare to the Persian language studies.

**Figure 4:**
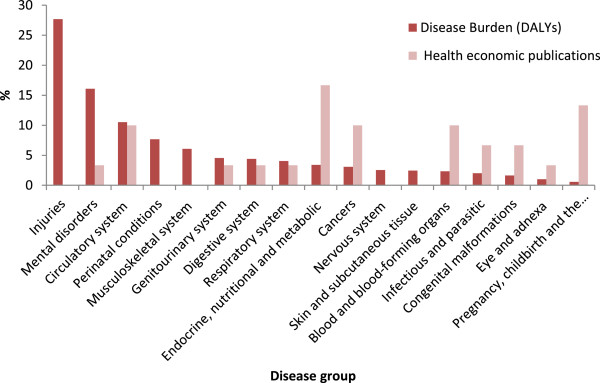
Comparison of the proportion of overall disease burden and the proportion of full economic evaluation publications in Iran since 1999.

Figure 4 shows the distribution of full economic evaluation studies by disease category, compared to the burden of disease in the country. Based on the figure, the five major areas of health problems in Iran are injuries (with 28% of DALYs), mental health (with 16% of DALYs), circulatory system diseases (with 11% of DALYs), perinatal conditions (with 8% of DALYs) and musculoskeletal system diseases (with 6% of DALYs). There are no economic evaluation studies for the three disease categories with the highest burdens, namely, injuries, perinatal conditions and musculoskeletal system diseases. Moreover, only one study investigated mental disorders, which accounted for 16% of burden of disease in the country. The most common diseases category covered by the economic evaluation studies are “Endocrine, nutritional and metabolic diseases” (n = 5, 17%, in this group Congenital hypothyroidism was investigated by four studies) and “Pregnancy, childbirth and the puerperium” (n = 4, 13%). “Circulatory system diseases”, “Cancers” and “Blood and blood-forming organs diseases” were other disease groups covered by the studies (in each group, n = 3 or 10%).

## Discussion

High quality economic evaluation data on interventions targeting high burden of diseases are important to aid informed decision-making and resource allocation. This study enumerates the availability of economic evaluation studies and critically assesses the quality of those data in the Iranian context. The findings showed that the number of economic evaluation studies in Iran is limited. This is comparable with settings such as Bangladesh [60], Nigeria [26], Saudi Arabia [61], Zimbabwe [62], and lagging behind countries such as Thailand [29], South Korea [24], India [63] and South Africa [64]. The number of available studies remains very low compared with high-income countries where economic evaluation is a relatively well-established and formal part of the policy making process [21,65]. The findings from this review also showed that many published Iranian economic evaluation studies did not meet current international standards and were of sub-optimal quality.

The review showed that CEA was the most frequently published economic evaluation in Iran. The relative ease of obtaining effectiveness data in the form of intermediate outcomes or natural units, and the application of arguably more straightforward computational methods in these types of evaluations [60] are two features that make CEA a practical method of analysis in many settings. This approach may be particularly attractive, when collecting comprehensive outcome measures such as DALYs and QALYs are time intensive and expensive to collect. CEA is useful in situations where outcome measures of interventions under investigation are similar. However, since the outcome measure may differ for different interventions, CEA can seldom be used to make comparisons across a broad set of interventions [1]. Taking into account this limitation, CUA or CBA arguably provide better tools for policy makers allocating resources across different health care programmes or even across different sectors [1].

Considering resource constraints, it would be expected that economic evaluations focus on interventions for diseases that have a significant impact on population health. However, the findings of the current review showed that the majority of economic evaluation studies concentrate on a small number of disease categories which do not include some of Iran’s major health problems. For example, injuries and mental disorders, two disease categories which contribute more than 40% to burden of disease in the country [6] and are likely to increase in the future [6], are strongly underrepresented in the Iranian economic evaluation literature. In addition, the findings show that researchers have paid a great deal of attention to curative interventions, while relatively little attention has been given to preventive interventions and care delivery strategies. These findings are consistent with previous studies [2,28,29]. A mismatch of economic evaluations with disease burden does not necessarily indicate inefficient resource allocation. It is possible that studies do not focus on high burden illnesses because there are few effective interventions for some health conditions. It is also possible that there exist a number of well-established, cost-effective interventions for some disease categories and as such new work in the area is not required. Difficulties in obtaining data on the effectiveness of interventions in some areas, such as mental disorders, can be another barrier to the conduct of evaluations for high burden illnesses. The interest of researchers and funding agencies could also play a role as the majority of research in Iran is funded by universities or other governmental research centres. Multiple criteria including equity and social justice are frequently used to determining health priorities and the burden of disease is only one of those criteria [2,28,66]. Nonetheless, we believe that comparing the priorities reflected in the evidence regarding the burden of disease, with those reflected in the economic evaluation literature in Iran can provide a useful starting point for discussing future priorities for economic evaluation research.

The review indicated that the Iranian economic evaluation studies had low adherence to good practice criteria for the reporting of economic evaluations and suffer from several methodological flaws. The perspective of an economic evaluation study is an important issue as it affects the measurement of both costs and outcomes of interventions. Yet few of the reviewed studies (only 23%) specified their perspective. This suggests that many authors are unaware of the importance of the perspective adopted and its effect on the costs and outcomes. Among the studies which stated their perspective, many failed to include all appropriate costs associated with their chosen perspective. For example, two studies [30,58] stated their perspective as societal but they didn’t measure indirect costs associated with their interventions and those studies that had taken a healthcare system or provider as their perspective, failed to include capital or equipment costs and some recurrent costs (e.g. overhead costs). In the context of Iran, obtaining data to estimate capital and overhead costs, particularly in hospital settings where most of the reviewed studies are based, might be difficult. Moreover, some part of these costs is usually incorporated in the inpatients and outpatients’ costs or hospital bills, which many of the reviewed studies included in their analysis. Using hospital charges/bills (and also health care tariffs) in economic evaluation have been criticised since they may not reflect the actual costs [1,67]. In the context of Iran in particular, hospital charges do not reflect true hospital costs due to government subsidies for hospital services and medicines [68]. Moreover, the effects of adverse events associated with interventions on use of resources and outcomes were also rarely [37,53] included in the reviewed studies. Lack of transparency in reporting intervention costs, was another important shortcoming of the reviewed studies. In some studies [31,40,43,44,55] it becomes impossible to ascertain what authors had actually done. Many studies failed to describe clearly how they measured and valued costs and did not provide details on type and quantities of resources and their price, which limits the possibility of replicating the evaluation in other settings.

Moreover, few studies calculated and reported ICER. Instead of an ICER, some studies reported the average cost-effectiveness ratio (i.e. total cost divided by total effect for the interventions being compared), which can flaw the conclusion of the evaluation and limit direct comparison between interventions. This is because an average ratio implies the comparison of each alternative with a hypothetical intervention with no costs and no effects [69].

A major weakness of the economic evaluation studies in the Iranian setting was the limited use of sensitivity analysis to explore the effect of uncertainty on findings. Only 27% of studies performed some sort of sensitivity analysis. Sensitivity analysis helps to assess reliability of the findings for the context of the study and can also facilitate consideration of the generalisability of findings to other settings [70]. Moreover, the review also showed that very few Iranian economic evaluation studies discounted costs and/or outcomes when the study period was more than one year. None of the studies provide justification for the discount rate used, even if that rate was zero, and none performed a sensitivity analysis on the rate used.

Low adherence to good practice for the conduct of economic evaluations and the other methodological shortcomings discussed above are not unique to the Iranian setting. Systematic reviews of economic evaluation studies in some other settings (both developed and developing countries) have reported similar shortcomings [21,23,24,26,29,60-65]. For this reason, many countries have developed formal and informal guidelines to standardize and improve the quality of economic evaluation in health care [71].

### Limitations of the review

Admittedly, this review may suffer from some limitations. This study included only published literature in peer-reviewed journals and excluded grey literature such as government reports, pharmaceutical company reports, academic theses and conference proceedings. The inclusion of only published literature might have introduced publication bias, since studies with positive results are more likely to be published than studies with negative findings [72-74]. In addition, although the database used for searching the studies published in Persian consisted of the majority of journals published in Iran, some journals or studies may not have been included in this database. Furthermore, as in any review study, it is difficult to rule out selection bias or disagreement between the criteria of the reviewers. To minimise this bias, we used pre-defined inclusion criteria and discussion of disagreement between the investigators throughout of the review process.

## Conclusion

The findings of this review indicate that the literature on economic evaluation in Iran is still at an early stage and these evaluations suffer from significant methodological flaws. Furthermore, the review reveals that economic evaluation studies have not focused on Iran’s most significant health problems i.e. those contributing most to the country’s burden of disease. Although interest in using inputs from economic evaluation and HTA studies has increased in Iran, evidence still points to scarce demand for and utilisation of these inputs by policy makers. This itself might undermine incentives to improve the quality of economic evaluation studies.

The findings from this study suggest that Iranian evaluations might benefit from the establishment of clear national guidelines on the conduct of economic evaluations. In addition, capacity building of local scientists in the conduct of economic evaluations may be a priority area in the future. This would require additional investment in the teaching of economic evaluation in Iranian universities and the training of health professionals and policy makers in the use of economic data. Moreover, priority areas for future economic evaluation should be established by collaboration between researchers across disciplines, and in communication with policy makers – taking explicit account of the national burden of disease.

## Abbreviations

GNI: Gross national income; GDP: Gross domestic production; MOHME: Ministry of health and medical education; MWSS: Ministry of Welfare and Social Security; EED: Economic evaluation database; SID: Scientific information database; CMA: Cost minimization analysis; CEA: Cost-effectiveness analysis; CUA: Cost-utility analysis; CBA: Cost-benefit analysis; WHO: World Health Organization; NBD: National burden of disease; RCT: Randomized controlled Trials; DALY: Disability-adjusted Life Years; QALY: Quality-adjusted Life Years; ICER: incremental cost-effectiveness ratio; BCR: Benefit-cost ratios; NPV: Net present value.

## Competing interests

The authors declare that they have no competing interests. No funding has been received for the conduct of this study and/or preparation of this manuscript.

## Authors’ contributions

HHB and AAK were involved in the study conception and design, literature search, designing checklist, data analysis, interpretation of the data, and writing the manuscript. JSW was involved in the study design, results interpretation, and finalization of the manuscript. All authors read and approved the final manuscript.

## Supplementary Material

Additional file 1 Search syntax. Click here for file

Additional file 2: Table S1 Summary characteristics of the studies reviewed (n = 30). Click here for file

Additional file 3: Table S2 Assessing quality of the studies reviewed using the criteria for good reporting of economic evaluations. Click here for file

## References

[B1] Drummond MF Sculpher MJ Torrance GW O’Brien BJ Stoddart GL Methods for the Economic Evaluation of Health Care Programmes 2005 Third edition Oxford: Oxford University Press

[B2] Catala-Lopez F Garcia-Altes A Alvarez-Martin E Genova-Maleras R Morant-Ginestar C Parada A Burden of disease and economic evaluation of healthcare interventions: are we investigating what really matters? BMC Health Serv Res 2011 11 75 21489236 10.1186/1472-6963-11-75 PMC3097252

[B3] World Health Organization Country profile of Iran 2014 WHO Available at: http://www.who.int/countries/irn/en/ (Accessed Jan 20, 2014)

[B4] Mehrdad R Health System in Iran The Japan Medical Association Journal (JMAJ) 2009 52 1 69 73

[B5] Davari M Haycox A Walley T The Iranian health insurance system; past experiences, present challenges and future strategies Iran J Public Health 2012 41 9 1 9 23193499 PMC3494208

[B6] Naghavi M Abolhassani F Pourmalek F Lakeh M Jafari N Vaseghi S Mahdavi Hezaveh N Kazemeini H The burden of disease and injury in Iran 2003 Popul Health Metr 2009 7 9 19527516 10.1186/1478-7954-7-9 PMC2711041

[B7] Shadpour K Health sector reform in Islamic Republic of Iran Hakim Research Journal 2006 9 3 1 18

[B8] AhmadKiadaliri A Haghparast-Bidgoli H Zarei A Measuring Efficiency of General Hospitals in the South of Iran World Appl Sci J 2011 13 6 1310 1316

[B9] World Bank Islamic Republic of Iran, Health Sector Review, Volume II: Background Sections 2007 Human Development Sector, Middle East and North Africa: The World Bank Group

[B10] Davari M Haycox A Walley T Health Care Challenges in Iran Iranian Journal of Public Health 2005 34 sup 30 31

[B11] Ibrahimipour H Maleki MR Brown R Gohari M Karimi I Dehnavieh R A qualitative study of the difficulties in reaching sustainable universal health insurance coverage in Iran Health Policy Plan 2011 26 6 485 495 21303879 10.1093/heapol/czq084

[B12] Palesh M Tishelman C Fredrikson S Jamshidi H Tomson G Emami A We noticed that suddenly the country has become full of MRI. Policy makers’ views on diffusion and use of health technologies in Iran Health Res Policy Syst 2010 8 9 20370906 10.1186/1478-4505-8-9 PMC2907640

[B13] Doherty J Kamae I Lee KKC Li H Li SC Liu GG Tarn YH Yang BM What is next for pharmacoeconomics and outcomes research in Asia? Value Health 2004 7 2 118 132 15164802 10.1111/j.1524-4733.2004.72330.x

[B14] Doaee SH Olyaeemanesh A Emami SH Mobinizadeh M Abooee P Nejati M Zolani GS Development and Implementation of Health Technology Assessment: A Policy Study Iranian Journal of Public Health 2013 42 50 54 23865016 PMC3712594

[B15] Palesh M Fredrikson S Jamshidi H Jonsson PM Tomson G Diffusion of magnetic resonance imaging in Iran Int J Technol Assess Health Care 2007 23 2 278 285 17493315 10.1017/S0266462307070377

[B16] Jefferson T Demicheli V Vale L Quality of systematic reviews of economic evaluations in health care Jama 2002 287 21 2809 2812 12038919 10.1001/jama.287.21.2809

[B17] Yothasamut J Tantivess S Teerawattananon Y Using economic evaluation in policy decision-making in Asian countries: mission impossible or mission probable? Value Health 2009 12 Suppl 3 S26 30 20586976 10.1111/j.1524-4733.2009.00623.x

[B18] Zarnke KB Levine MA O'Brien BJ Cost-benefit analyses in the health-care literature: don't judge a study by its label J Clin Epidemiol 1997 50 7 813 822 9253393 10.1016/s0895-4356(97)00064-4

[B19] Drummond MF Jefferson TO Guidelines for authors and peer reviewers of economic submissions to the BMJ. The BMJ Economic Evaluation Working Party BJM 1996 313 7052 275 283 10.1136/bmj.313.7052.275 PMC2351717 8704542

[B20] Evers S Goossens M de Vet H van Tulder M Ament A Criteria list for assessment of methodological quality of economic evaluations: Consensus on Health Economic Criteria Int J Technol Assess Health Care 2005 21 2 240 245 15921065

[B21] Dalziel K Segal L Mortimer D Review of Australian health economic evaluation - 245 interventions: what can we say about cost effectiveness? Cost Eff Resour Alloc 2008 6 9 18489788 10.1186/1478-7547-6-9 PMC2413209

[B22] Neumann PJ Stone PW Chapman RH Sandberg EA Bell CM The quality of reporting in published cost-utility analyses, 1976–1997 Ann Intern Med 2000 132 12 964 972 10858180 10.7326/0003-4819-132-12-200006200-00007

[B23] Cooper N Coyle D Abrams K Mugford M Sutton A Use of evidence in decision models: an appraisal of health technology assessments in the UK since 1997 J Health Serv Res Policy 2005 10 4 245 250 16259692 10.1258/135581905774414187

[B24] Lee KS Brouwer WB Lee SI Koo HW Introducing economic evaluation as a policy tool in Korea: will decision makers get quality information? a critical review of published Korean economic evaluations Pharmacoeconomics 2005 23 7 709 721 15987227 10.2165/00019053-200523070-00005

[B25] Schwappach DL Boluarte TA HEE-GER: a systematic review of German economic evaluations of health care published 1990–2004 BMC Health Serv Res 2007 7 7 17222334 10.1186/1472-6963-7-7 PMC1781069

[B26] Gavaza P Rascati KL Oladapo AO Khoza S The state of health economic evaluation research in Nigeria: a systematic review Pharmacoeconomics 2010 28 7 539 553 20550221 10.2165/11536170-000000000-00000

[B27] Murray C Lopez A The Global Burden Of Diseases: A Comprehensive Assessment Of Mortality And Disability From Diseases, Injuries And Risk Factors In 1990 And Projected To 2020 1996 WHO and World Bank: Harvard School of Public Health

[B28] Neumann PJ Rosen AB Greenberg D Olchanski NV Pande R Chapman RH Stone PW Ondategui-Parra S Nadai J Siegel JE Weinstein MC Can we better prioritize resources for cost-utility research? Med Decis Making 2005 25 4 429 436 16061895 10.1177/0272989X05276853

[B29] Teerawattananon Y Russell S Mugford M A systematic review of economic evaluation literature in Thailand: are the data good enough to be used by policy-makers? Pharmacoeconomics 2007 25 6 467 479 17523752 10.2165/00019053-200725060-00003

[B30] Adibi P Rezailashkajani M Roshandel D Behrouz N Ansari S Somi MH Shahraz S Zali MR An economic analysis of premarriage prevention of hepatitis B transmission in Iran BMC Infect Dis 2004 4 31 15347430 10.1186/1471-2334-4-31 PMC517713

[B31] Aslanabadi S Ghalehgolab-Behbahan A Zarrintan S Jamshidi M Seyyedhejazi M Transanal one-stage endorectal pull-through for Hirschsprung's disease: a comparison with the staged procedures Pediatr Surg Int 2008 24 8 925 929 18512060 10.1007/s00383-008-2186-9

[B32] Nakhaee N Mirahmadizadeh AR Gorji HA Mohammadi M Assessing the cost-effectiveness of contraceptive methods in Shiraz, Islamic Republic of Iran East Mediterr Health J 2002 8 1 55 63 15330561

[B33] Azizi F Ataie L Hedayati M Mehrabi Y Sheikholeslami F Effect of long-term continuous methimazole treatment of hyperthyroidism: comparison with radioiodine Eur J Endocrinol 2005 152 5 695 701 15879354 10.1530/eje.1.01904

[B34] Allameh Z Davari M Emami MH Cost-effectiveness analysis of colorectal cancer screening methods in Iran Arch Iran Med 2011 14 2 110 114 21361717

[B35] Yaghoubi M Aghayan HR Arjmand B Emami-Razavi SH Cost-effectiveness of homograft heart valve replacement surgery: an introductory study Cell Tissue Bank 2011 12 2 153 158 19949875 10.1007/s10561-009-9165-9

[B36] Daliri AA Haghparast H Mamikhani J Cost-effectiveness of prophylaxis against on-demand treatment in boys with severe hemophilia A in Iran Int J Technol Assess Health Care 2009 25 4 584 587 19845990 10.1017/S0266462309990420

[B37] Bastani P Kiadaliri AA Cost-Utility Analysis of Adjuvant Therapies for Breast Cancer in Iran Int J Technol Assess Health Care 2012 28 2 110 114 22559752 10.1017/S0266462312000049

[B38] Rasekh HR Imani A Karimi M Golestani M Cost-utility analysis of immune tolerance induction therapy versus on-demand treatment with recombinant factor VII for hemophilia A with high titer inhibitors in Iran Clinicoecon Outcomes Res 2011 3 207 212 22163168 10.2147/CEOR.S25909 PMC3234155

[B39] Shamshiri AR Yarahmadi S Forouzanfar MH Haghdoost AA Hamzehloo G Naieni KH Evaluation of Current Guthrie TSH Cut-off Point in Iran Congenital Hypothyroidism Screening Program: A Cost-Effectiveness Analysis Arch Iran Med 2012 15 3 136 141 22369300

[B40] Gholipour C Shalchi RA Abassi M Efficacy and safety of early laparoscopic common bile duct exploration as primary procedure in acute cholangitis caused by common bile duct stones J Laparoendosc Adv Surg Tech A 2007 17 5 634 638 17907977 10.1089/lap.2006.0199

[B41] Delavari AR Yarahmadi SH Birjandi R Mahdavi AR Norouzi Nejad A Dini M Cost-Benefit Analysis of the Neonatal Screening Program Implementation for Congenital Hypothyroidism in I. R. Iran Int J Endocrinol Metab 2006 4 2 84 87

[B42] Yarahmadi SH Tabibi SJ Alimohammadzadeh KH Ainy E Gooya MM Mojarrad M Delgoshaei B Cost–Benefit and Effectiveness of Newborn Screening of Congenital Hypothyroidism: Findings from a National Program in Iran Int J Endocrinol Metab 2010 8 1 1 6

[B43] Shafiei A AliMohamadi S Zamani M Nasrollahi S Radnia N Maternal and fetal complications with expecting treatment and labour induction after 40th week of gestational age The Journal of Qazvin University of Medical Sciences 2003 28 Autumn Supplement 77 80

[B44] Abolghasemi H Eshghi P Rahiminejhad S Hatami S Evaluation and cost-effectiveness of prevention program of major Thalassemia in Sistan & Balouchestan and Fars provinces Hakim 2006 8 4 8 14

[B45] Sharifi F Ghasemi S Abniki M Nazari N Evaluating effectiveness and cost-benefit of influenza vaccination among Kahrizak Elderly Nursing Home Residents Salmand 2007 2 5 370 378

[B46] Memarian R Mohammadi E Armat MR Compare complications, costs and direct care of the heparin lock procedure and the KVO procedure Journal of Zanjan University of Medical Sciences 1999 7 28 48 54

[B47] Farajzadegan Z Mirmoghtadaee P Mehrabian F Screening of asymptomatic bacteriuria: Urinalysis or Urine culture? Which one is more cost- effective? Journal of Isfahan Medical School 2008 26 89 119 126

[B48] Riahi L Tofighi S Ghanbari-Nikoo A The Cost-benefit Analysis on Screening Congenital Hypothyroeidism in Qazvin City live Born in 2008 Journal of Medical Council of Islamic Republic of Iran 2012 30 1 96 103

[B49] Moafi A Soheilipoor F Amini A Beheshti M Comparing efficacy and side effects of Pd-Grastim and Neupogen for prevention of neutropenia after chemotherapy in children Iran J Pediatr 2006 16 2 143 148

[B50] Lotfalizadeh M Teymoori M Comparison of efficacy and safety of nifedipine versus magnesium sulfatein treatment of preterm labor Iraninan Journal of Obstetrics Gynecology and Infertility 2009 13 2 7 12

[B51] Poorsadegh M Khadivi E Rezaei S Sagheb-Hosseinpoor S Gharaei SH Babaeyan M The Comparative study of two treatment protocols in short course control of symptoms of allergic rhinitis patients The Iranian Journal of Otorhinolaryngology 2007 19 48 71 77

[B52] Karimi I Zohour AR Vianchi A Cost-effectiveness analysis of dialysis and kidney transplants using DALY in Shahid Hasheminejad Hospital Iran Journal of Health Administration 2005 8 19 45 49

[B53] Nasiri A Nemat R Arshadi FS AliMohammadpoor R Comparing costs and complications of general anesthesia and spinal anesthesia in surgery of patients above 55 years old in Mazandaran University of Medical Sciences Journal of Mazandaran University of Medical Sciencess 2006 16 51 59 69

[B54] Karimi-Aghdam MA Samadi M Ghafari S Mahmoudpoor F Comparing treatment outcomes, complications and costs of occlusion of Patent Ductus Arteriosus (PDA) with nonsurgical versus surgical methods Journal of Ardabil University of Medical Sciences 2008 18 2 172 178

[B55] Arab M Comparing complications and costs of normal delivery or caesarean after previous caesarean Scientific Journal of Hamadan University of Medical Sciences 2001 8 2 29 33

[B56] Ghazizadeh A Cost-benefit of treating depressed patients in Kurdistan primary health care system Scientific Journal of Kurdistan University of Medical Sciences 2001 5 19 14 16

[B57] Behradmanesh S Dehghan H Tavasoli AA Sarafzadeghan N Sadeghi M Najafian J Habibi HR Comparing costs of methods for early diagnosis of acute and non-typical chest pain with non-diagnostic ECG Journal of Isfahan Medical School 2002 20 67 26 32

[B58] Forouzanfar MH Fotohi A Majdzadeh SR Jamali P Comparing cost-effectiveness of amblyopia screening by optometrists and trained kindergarten staff The quarterly Journal of the School of Public Health and Institute of Public Health Research 2008 6 3–4 87 99

[B59] MirMohammad Sadeghi M Masaeli Z Jaberi MR A comparison of bloodless and classic methods of coronary artery bypass grafting in Chamran hospital Isfahan The Scientific Journal of Iranian Blood Transfusion 2004 1 1 51 58

[B60] Hoque ME Khan JA Hossain SS Gazi R Rashid HA Koehlmoos TP Walker DG A systematic review of economic evaluations of health and health-related interventions in Bangladesh Cost Eff Resour Alloc 2011 9 12 21771343 10.1186/1478-7547-9-12 PMC3158529

[B61] Al-Aqeel SA State of health economic evaluation research in Saudi Arabia: a review Clinicoecon Outcomes Res 2012 4 177 184 22826634 10.2147/CEOR.S31087 PMC3401052

[B62] Gavaza P Rascati K Brown C Lawson K Mann T The state of health economic and pharmacoeconomic evaluation research in Zimbabwe: A review Curr Ther Res Clin E 2008 69 3 268 285 10.1016/j.curtheres.2008.06.005 PMC3969967 24692805

[B63] Desai PR Chandwani HS Rascati KL Assessing the quality of pharmacoeconomic studies in India: a systematic review Pharmacoeconomics 2012 30 9 749 762 22720697 10.2165/11590140-000000000-00000

[B64] Gavaza P Rascati KL Oladapo AO Khoza S The state of health economic research in South Africa: a systematic review Pharmacoeconomics 2012 30 10 925 940 22809450 10.2165/11589450-000000000-00000

[B65] Drummond MF Iglesias CP Cooper NJ Systematic reviews and economic evaluations conducted for the National Institute for Health and Clinical Excellence in the United Kingdom: a game of two halves? Int J Technol Assess Health Care 2008 24 2 146 150 18400116 10.1017/S0266462308080203

[B66] Jamison DT Breman JG Measham AR Alleyne G Claeson M Evans DB Jha P Mills A Musgrove PH Disease Control Priorities in Developing Countries 2006 Washington DC: World Bank 21250309

[B67] Brouwer W Rutten F Koopmanschap M Drummond M, McGuire A Costing in economic evaluations Economic Evaluation in Health Care: Merging Theory With Practice 2001 Oxford: Oxford University Press 68 93

[B68] Haghparast-Bidgoli H Saadat S Bogg L Yarmohammadian MH Hasselberg M Factors affecting hospital length of stay and hospital charges associated with road traffic-related injuries in Iran BMC Health Serv Res 2013 13 281 23875993 10.1186/1472-6963-13-281 PMC3726419

[B69] Drummond M Sculpher M Common methodological flaws in economic evaluations Med Care 2005 43 7 Suppl 5 14 16056003 10.1097/01.mlr.0000170001.10393.b7

[B70] Walker D Fox-Rushby JA Economic evaluation of communicable disease interventions in developing countries: a critical review of the published literature Health Econ 2000 9 8 681 698 11137950 10.1002/1099-1050(200012)9:8<681::aid-hec545>3.0.co;2-x

[B71] Hjelmgren J Berggren F Andersson F Health economic guidelines–similarities, differences and some implications Value Health 2001 4 3 225 250 11705185 10.1046/j.1524-4733.2001.43040.x

[B72] Freemantle N Mason J Publication bias in clinical trials and economic analyses Pharmacoeconomics 1997 12 1 10 16 10169384 10.2165/00019053-199712010-00002

[B73] Bell CM Urbach DR Ray JG Bayoumi A Rosen AB Greenberg D Neumann PJ Bias in published cost effectiveness studies: systematic review BMJ 2006 332 7543 699 703 16495332 10.1136/bmj.38737.607558.80 PMC1410902

[B74] Hillman AL Eisenberg JM Pauly MV Bloom BS Glick H Kinosian B Schwartz JS Avoiding bias in the conduct and reporting of cost-effectiveness research sponsored by pharmaceutical companies N Engl J Med 1991 324 19 1362 1365 1901959 10.1056/NEJM199105093241911

