# Supplementary material for: Do economic evaluation studies inform effective healthcare resource allocation in Iran? A critical review of the literature
Source: Cost Eff Resour Alloc. 2014 Jul 11;12:15. doi: 10.1186/1478-7547-12-15 (PMC4105166; doi:10.1186/1478-7547-12-15)
Supplement: Additional file 1 — Search syntax. [file 1478-7547-12-15-S1.docx]

Pubmed/MEDLINE

"cost-minimization"[Title/Abstract] OR "cost-utility"[Title/Abstract] OR "cost-effectiveness"[Title/Abstract] OR "cost-benefit"[Title/Abstract] OR "economic evaluation"[Title/Abstract] OR "cost-saving"[Title/Abstract] OR "cost analysis" [Title/Abstract] AND "Iran*"[Title/Abstract]

Web of Science

TOPIC: (((("cost-minimization" or "cost-utility" or "cost-effectiveness" or "cost-benefit" or "economic evaluation" or "cost-saving" or "cost analysis") and "Iran")))

Refined by: RESEARCH AREAS=( HEALTH CARE SCIENCES SERVICES OR LIFE SCIENCES BIOMEDICINE OTHER TOPICS OR PUBLIC ENVIRONMENTAL OCCUPATIONAL HEALTH OR PHARMACOLOGY PHARMACY OR NURSING OR PEDIATRICS OR NUTRITION DIETETICS OR GASTROENTEROLOGY HEPATOLOGY OR INFECTIOUS DISEASES OR OPHTHALMOLOGY OR SURGERY OR ANESTHESIOLOGY OR BIOCHEMISTRY MOLECULAR BIOLOGY OR DENTISTRY ORAL SURGERY MEDICINE OR RESEARCH EXPERIMENTAL MEDICINE OR REPRODUCTIVE BIOLOGY OR GENERAL INTERNAL MEDICINE OR PSYCHOLOGY OR ENDOCRINOLOGY METABOLISM OR DERMATOLOGY OR ONCOLOGY OR OBSTETRICS GYNECOLOGY OR MEDICAL INFORMATICS OR MICROBIOLOGY OR ORTHOPEDICS OR MEDICAL LABORATORY TECHNOLOGY OR CARDIOVASCULAR SYSTEM CARDIOLOGY OR RADIOLOGY NUCLEAR MEDICINE MEDICAL IMAGING OR RESPIRATORY SYSTEM OR REHABILITATION OR HEMATOLOGY OR PATHOLOGY OR TROPICAL MEDICINE OR NEUROSCIENCES NEUROLOGY ) AND COUNTRIES/TERRITORIES=( IRAN ) AND DOCUMENT TYPES=( ARTICLE )

Embase (via OvidSP)

(("cost-minimization" or "cost-utility" or "cost-effectiveness" or "cost-benefit" or "economic evaluation" or "cost-saving" or "cost analysis") and "Iran*").m_titl.

Limit to human

EconLit

(("cost-minimization" or "cost-utility" or "cost-effectiveness" or "cost-benefit" or "economic evaluation" or "cost-saving" or "cost analysis") and "Iran")

**Search modes** - Boolean/Phrase

NHS Economic Evaluation Database (EED)

(((cost-minimization or cost-utility or cost-effectiveness or cost-benefit or economic evaluation or cost-saving or cost analysis) and Iran))
