# Supplementary material for: Do economic evaluation studies inform effective healthcare resource allocation in Iran? A critical review of the literature
Source: Cost Eff Resour Alloc. 2014 Jul 11;12:15. doi: 10.1186/1478-7547-12-15 (PMC4105166; doi:10.1186/1478-7547-12-15)
Supplement: Additional file 2: Table S1 — Summary characteristics of the studies reviewed (n = 30). [file 1478-7547-12-15-S2.docx]

| **Primary Author and publication year** | **Type of economic evaluation** | **Study design** | **Perspective (as stated by authors)** | **Time horizon** | **Outcome description** | **Disease investigated** | **Level of care/ intervention type** | **Type and source of data used** | **Sample size** | **Primary source of funding** |
| --- | --- | --- | --- | --- | --- | --- | --- | --- | --- | --- |
| Adibi et al, 2004 | CEA | Modelling (Decision tree) | Mixed (Health care system and society) | 10 years | Number of chronic HBV infections averted | Hepatitis B | Screening | Secondary (Costs and epidemiological data collected from Iranian sources. Effectiveness data collected from settings other than Iran) | Not specified | Government (Medical University) |
| Aslanabadi et al, 2008 | CEA | RCT | Not specified | 18 months | Average number of Surgical complications | Hirschsprung’s disease | Curative (Surgery) | Primary | 42 children (35 boys and 7 girls between 3 days and 12 years of age) | Not listed |
| Nakhaee et al, 2002 | CEA | Observational (Cross-Sectional) | Not specified | 12 months | Conventional couple-years of protection (CYP), and adjusted CYPs. | Contraceptive | Primary prevention | Secondary (Cost data collected from local and national sources. Effectiveness obtained from international sources) | 4550 contraceptive users (Different samples for each contraceptive method) | Government (Medical University) |
| Azizi et al, 2005 | CEA | RCT | Health care system | 10 years | Primary outcome measure: numbers of occurrences of thyroid dysfunction (Several physiological and functional indicators were measured including quality of life). | Hypothyroidism | Curative (Pharmaceutical) | Primary | 504 patients | Not listed |

Table S1: Summary characteristics of the reviewed studies (n=30)

Table S1- continued

| **Primary Author and publication year** | **Type of economic evaluation** | **Study design** | **Perspective (as stated by authors)** | **Time horizon** | **Outcome description** | **Disease investigated** | **Level of care/ intervention type** | **Type and source of data used** | **Sample size** | **Primary source of funding** |
| --- | --- | --- | --- | --- | --- | --- | --- | --- | --- | --- |
| Allameh Pharm et al, 2011 | CEA | Modelling (Type of model was not specified) | Not specified | 20 years | Number of patients detected (true positive patients) | Colorectal cancer | Screening | Mixed (Sensitivity and specificity of each method obtained through a literature review) | 100,000 hypothetical healthy adult aged 45 – 65 years old | Not listed |
| Yaghoubi et al, 2009 | CUA | Observational (Cross-Sectional) | Not specified | Not specified | QALYs gained (Quality of life was measured using the SF-36 questionnaire) | Damaged heart valves | Curative (Surgery) | Primary | 60 patients (40 men and 20 women) | Not listed |
| Ahmad Kia Daliri et al, 2009 | CEA | Observational (Cross-Sectional) | Third party | 6 months | Number of bleeding event prevented | Type A haemophilia | Curative (Pharmaceutical) | Primary | 25 type A hemophiliacs (boys 0–9 years old) | Iranian Ministry of Health |
| Bastani et al, 2012 | CUA | Quasi-experimental | Third party | 8 months | QALYs gained (Health-related quality of life was assessed using the European Organization for Research and Treatment of Cancer questionnaire [QLQ-C30]. QLQ-C30 scale scores were mapped to 15D and EQ 5D utilities to estimate QALYs) | Breast cancer | Curative (Pharmaceutical) | Primary | 100 patients (32 in TAC and 68 in FAC groups) | Government (Medical University) |
| Gholipour et al, 2007 | CEA | Observational (Prospective) | Not specified | Not specified | The main outcomes reported were mortality rate, complications of surgery, and the length of hospital and intensive care unit (ICU) stay. | Acute gallstone cholangitis | Curative (Surgery) | Primary | 73 patients (37 in LCBDE arm and 36 in OCBDE) | Not listed |

Table S1- continued

| **Primary Author and publication year** | **Type of economic evaluation** | **Study design** | **Perspective (as stated by authors)** | **Time horizon** | **Outcome description** | **Disease investigated** | **Level of care/ intervention type** | **Type and source of data used** | **Sample size** | **Primary source of funding** |
| --- | --- | --- | --- | --- | --- | --- | --- | --- | --- | --- |
| Rasekh et al, 2011 | CUA | Modelling (Type of model used was not specified) | Ministry of Health (health system) | 10 years | QALYs gained | Type A hemophilia | Curative (Pharmaceutical) | Mixed (Utilities/QALYs gained from each strategies were derived from a study in UK) | Not specified | Government (Medical University) |
| Shamshiri et al, 2012 | CUA | Modelling (Decision tree) | Caregiver/Health provider | Life time | DALYs | Congenital hypothyroidism (CH) | Screening | Secondary (Costs, outcome and other model parameters were derived from similar studies in Iran.) | A hypothetical cohort of 10,000 neonates (5,000 females and 5,000 males) | Government (Medical University) |
| Delavari et al, 2006 | CBA | Observational (Prospective) | Not specified | 14 years | The care cost of mentally retarded individuals (if screening not done) was calculated as benefit. The costs were estimated for 14 years- start when a child is 6 years old until s/he is 20 years old. | Congenital Hypothyroidism | Screening | Primary | 15,598 newborns | Not listed |
| Yarahmadi et al, 2010 | CBA | Observational (Prospective) | Not specified | Life time | Costs related to training and care of patients (plus Medical costs) suffering from mental retardation (if screening not done) were estimated for 70 years. | Congenital Hypothyroidism | Screening | Primary | 1,165,169 newborns (who were screened across whole country during 2008) | Not listed |
| Ghazizadeh, 2001 | CBA | Observational (Case-control) | Not specified | Not specified | Benefit was measured as economic savings due to reduction in hours cared by the families and also increased hours of working for the treated patients. | Depression | Mode of service delivery | Primary | 117 patients (57 case and 60 control) | Not listed |

Table S1- continued

| **Primary Author and publication year** | **Type of economic evaluation** | **Study design** | **Perspective (as stated by authors)** | **Time horizon** | **Outcome description** | **Disease investigated** | **Level of care/ intervention type** | **Type and source of data used** | **Sample size** | **Primary source of funding** |
| --- | --- | --- | --- | --- | --- | --- | --- | --- | --- | --- |
| Farajzadegan et al, 2008 | CEA | RCT | Not specified | Not specified | Prevented symptomatic bacteriuria | Asymptomatic bacteriuria (ABU) during pregnancy | Screening | Primary | 200 pregnant women (100 in each arm) | Not listed |
| Abolghasemi et al, 2006 | CEA | Modelling (Decision tree--before-after study) | Not specified | 4 years | Primary outcome: prevented birth of child with Thalassemia major. Secondary outcome: marriage dissuation rate | Thalassemia | Screening | Primary | Not specified | Government (Ministry of Health) |
| Nasiri et al, 2006 | CEA | Observational (Cross-Sectional) | Not specified | Not specified | Number of complications after surgery | Other(no disease) | Curative (Medical procedure) | Primary | 120 elderly patients (80 in GA and 40 in SA) | Government (Medical University) |
| Sharifi et al, 2007 | CEA | Quasi-experimental | Not specified | 10 months | Incidence of Influenza-like illness as the Primary outcome and total mortality rate as the Secondary outcome. | Influenza | Primary preventive (vaccination) | Primary | 941 elderly (389 in experiment and 557 in control arms) | Not listed |
| Karimi Aghdam et al, 2008 | CEA | Observational (Cross-Sectional) | Not specified | 6 months | Number of complications | Patent Ductus Arteriosus (PDA) | Curative (Surgery) | Primary | 63 patients (42 in Surgical and 21 in nonsurgical groups) | Not listed |
| Arab, 2001 | CEA | Observational (Cross-Sectional) | Not specified | Not specified | Number of complications | Delivery | Curative (Surgery) | Primary | 352 (81 in Normal delivery and 271 in caesarean groups) | Not listed |

Table S1- continued

| **Primary Author and publication year** | **Type of economic evaluation** | **Study design** | **Perspective (as stated by authors)** | **Time horizon** | **Outcome description** | **Disease investigated** | **Level of care/ intervention type** | **Type and source of data used** | **Sample size** | **Primary source of funding** |
| --- | --- | --- | --- | --- | --- | --- | --- | --- | --- | --- |
| Karimi et al, 2005 | CUA | Observational (Cross-Sectional) | Not specified | Not specified | DALYs | Kidney failure | Curative (Surgery) | Primary | Not specified | Not listed |
| Forouzanfar et al, 2008 | CEA | Modelling (Decision tree) | Societal | Not specified | Number of diagnosed patient | Amblyopia | Screening | Secondary (both costs and outcome data were extracted from a pilot study done by the authors) | A hypothetical cohort of 1000 kindergarten children | Not listed |
| Behradmanesh et al, 2002 | CEA | RCT | Not specified | 2 months | Primary outcome: quality of life scores | Acute and non-typical chest pain | Diagnostic | Primary | 202 patients | Not listed |
| Shafiei et al, 2003 | CEA | RCT | Not specified | Not specified | Several outcome measures were compared: infant convulsion,  fetal mortality rate, rate of caesarean section, birth weight, etc. | Delivery after 40th week of gestational age | Curative (Medical procedure) | Primary | 108 women (48 in EI and 60 in EM arms) | Not listed |
| Poorsadegh et al, 2007 | CEA | RCT | Not specified | 45 days | Reduction in the symptoms of Allergic rhinitis | Allergic rhinitis | Curative (Pharmaceutical) | Mixed (Cost of treatment/medication in each protocol were obtained from the local sources) | 65 patients (30 under first protocol and 35 under the second) | Not listed |
| MirMohammad Sadeghi et al, 2004 | CEA | Observational (Cross-Sectional) | Not specified | Not specified | Number of postoperative complications | Coronary Heart Disease | Curative (Surgery) | Primary | 200 patients (100 in each group) | Not listed |

Table S1- continued

| **Primary Author and publication year** | **Type of economic evaluation** | **Study design** | **Perspective (as stated by authors)** | **Time horizon** | **Outcome description** | **Disease investigated** | **Level of care/ intervention type** | **Type and source of data used** | **Sample size** | **Primary source of funding** |
| --- | --- | --- | --- | --- | --- | --- | --- | --- | --- | --- |
| Moafi et al, 2006 | CEA | RCT (Cross-over) | Not specified | Not specified | Episodes of  neutropenia and fever | Cancer | Curative (Pharmaceutical) | Mixed (Cost of treatment/medication were obtained from the local sources) | 60 children 1-15 years old (30 in each group) | Government (Medical University) |
| Lotfalizadeh et al, 2009 | CEA | RCT | Not specified | Not specified | Preterm labour prevented | Premature delivery | Curative (Pharmaceutical) | Primary | 80 pregnant women (40 in each group) | Government (Medical University) |
| Memarian et al, 1999 | CEA | Quasi-experimental (Cross-over) | Not specified | 72 hours | Complications rate and time spent for direct care | Other (no disease) | Curative (Medical procedure) | Primary | 30 patients | Not listed |
| Riahi et al, 2012 | CBA | Observational (Prospective) | Not specified | Life time | The care cost of mentally retarded individuals (if screening not done) was calculated as benefits for 70 years. | Congenital Hypothyroidism | Screening | Primary | 8456 newborns (in the province, who were screened during 2008) | Not listed |
