# Supplementary material for: Do economic evaluation studies inform effective healthcare resource allocation in Iran? A critical review of the literature
Source: Cost Eff Resour Alloc. 2014 Jul 11;12:15. doi: 10.1186/1478-7547-12-15 (PMC4105166; doi:10.1186/1478-7547-12-15)
Supplement: Additional file 3: Table S2 — Assessing quality of the studies reviewed using the criteria for good reporting of economic evaluations. [file 1478-7547-12-15-S3.docx]

Table S2: Assessing quality of the reviewed studies using the criteria for good reporting of economic evaluations (n=30)

| **First Author** | **Well defined questions in answerable form** | **Competing alternatives clearly described** | **Time horizon stated** | **Perspective specified** | **All important and relevant costs for each alternative identified** | **All included cost measured appropriately** | **All included costs valued appropriately** | **Sources of cost data included** | **Sources of outcome data included** | **ICER/ BCR/ NPV calculated and reported*** | **Cost discounted**** | **Outcome discounted**** | **Sensitivity analysis performed** | **Generasilibity of findings discussed** | **Funding sources disclosed** |
| --- | --- | --- | --- | --- | --- | --- | --- | --- | --- | --- | --- | --- | --- | --- | --- |
| Adibi et al, 2004 | Yes | Yes | Yes | Yes | No | Yes | Yes | Yes | Yes | No | Yes | No | Yes | Yes | Yes |
| Aslanabadi et al, 2008 | Yes | Yes | Yes | No | No | No | No | No | Yes | No | N/R | N/R | No | No | No |
| Nakhaee et al, 2002 | Yes | Yes | Yes | No | No | No | No | Yes | Yes | Yes (ICER) | N/R | N/R | Yes | No | Yes |
| Azizi et al, 2005 | Yes | No | Yes | Yes | No | No | No | Yes | Yes | No | No | No | No | No | No |
| Allameh Pharm et al, 2011 | Yes | No | Yes | No | Yes | Yes | Yes | Yes | Yes | No | No | No | No | No | No |
| Yaghoubi et al, 2009 | Yes | Yes | No | No | No | No | No | Yes | Yes | Yes | No | No | No | No | No |
| Ahmad Kia Daliri et al, 2009 | Yes | Yes | Yes | Yes | Yes | Yes | Yes | Yes | Yes | Yes (ICER) | N/R | N/R | Yes | No | Yes |
| Bastani et al, 2012 | Yes | Yes | Yes | Yes | Yes | No | Yes | Yes | Yes | No | N/R | N/R | Yes | Yes | Yes |
| Gholipour et al, 2007 | Yes | No | No | No | No | No | No | No | Yes | No | No | No | No | No | No |
| Rasekh et al, 2011 | Yes | Yes | Yes | Yes | No | Yes | Yes | No | Yes | Yes | No | No | Yes | No | Yes |
| Shamshiri et al, 2012 | Yes | Yes | Yes | Yes | Yes | Yes | Yes | Yes | Yes | Yes (ICER) | Yes | Yes | Yes | No | Yes |
| Delavari et al, 2006 | Yes | No | Yes | No | Yes | Yes | No | Yes | Yes | Yes (BCR) | No | No | No | No | No |
| Yarahmadi et al, 2010 | Yes | No | Yes | No | No | Yes | Yes | Yes | Yes | Yes (BCR) | Yes | Yes | No | No | No |
| Ghazizadeh, 2001 | Yes | No | No | No | No | No | No | Yes | Yes | No | No | No | No | No | No |
| Farajzadegan et al, 2008 | Yes | Yes | No | No | Yes | Yes | Yes | Yes | Yes | No | No | No | Yes | Yes | No |
| Abolghasemi et al, 2006 | Yes | Yes | Yes | No | No | No | No | Yes | Yes | No | No | No | No | No | Yes |
| Nasiri et al, 2006 | Yes | Yes | No | No | No | Yes | Yes | Yes | Yes | No | No | No | No | No | Yes |
| Sharifi et al, 2007 | Yes | No | Yes | No | Yes | Yes | Yes | Yes | Yes | No | N/R | N/R | No | No | No |
| Karimi Aghdam et al, 2008 | Yes | Yes | Yes | No | No | No | No | Yes | Yes | No | N/R | N/R | No | No | No |
| Arab, 2001 | Yes | No | No | No | No | No | No | Yes | Yes | No | No | No | No | No | No |
| Karimi et al, 2005 | Yes | No | No | No | No | No | No | Yes | Yes | No | No | No | No | No | No |
| Forouzanfar et al, 2008 | Yes | Yes | No | Yes | Yes | Yes | No | Yes | Yes | No | No | No | Yes | Yes | No |
| Behradmanesh et al, 2002 | Yes | Yes | Yes | No | Yes | Yes | No | Yes | Yes | No | N/R | N/R | No | No | No |
| Shafiei et al, 2003 | Yes | No | No | No | No | No | No | No | Yes | No | No | No | No | No | No |
| Poorsadegh et al, 2007 | Yes | No | Yes | No | No | Yes | Yes | No | Yes | No | N/R | N/R | No | No | No |
| MirMohammad Sadeghi et al, 2004 | Yes | Yes | No | No | No | No | No | Yes | Yes | No | No | No | No | No | No |
| Moafi et al, 2006 | Yes | Yes | No | No | No | No | No | Yes | Yes | No | No | No | No | No | Yes |
| Lotfalizadeh et al, 2009 | Yes | Yes | No | No | No | No | No | No | Yes | No | No | No | No | No | Yes |
| Memarian et al, 1999 | Yes | Yes | Yes | No | No | Yes | Yes | Yes | Yes | No | N/R | N/R | No | No | No |
| Riahi et al, 2012 | Yes | No | Yes | No | No | No | No | Yes | No | Yes (BCR) | No | Yes | No | No | No |

* ICER: Incremental Cost Effectiveness Analysis, BCR: Benefit-Cost ratio, NPV: Net Present Value

** N/R: Not relevant. Discounting cost and outcomes was applicable only for those studies with time horizon more than one year.
